# Sexual and reproductive life in adolescents and young adults with phenylketonuria: a cross-sectional study

**DOI:** 10.1186/s13023-026-04509-w

**Published:** 2026-07-20

**Authors:** Bożena Mikołuć, Dariusz Walkowiak, Dorota Korycińska – Chaaban, Łukasz Kałużny, Maria Giżewska, Ewa Starostecka, Agata Lange, Jarosław Walkowiak

**Affiliations:** 1 Department of Pediatrics, Rheumatology, Immunology and Metabolic Bone Diseases, Medical University of Bialystok, Białystok, Poland; 2 Department of Organization and Management in Health Care, Poznan University of Medical Sciences, Przybyszewskiego 39, Poznań, 60-356 Poland; 3 Children’s Memorial Health Institute, Warsaw, Poland; 4 Department of Pediatric Gastroenterology and Metabolic Diseases, Poznan University of Medical Sciences, Poznań, Poland; 5 Department of Pediatrics, Rare Diseases and Metabolic Medicine, Pomeranian Medical University in Szczecin, Szczecin, Poland; 6 Institute Centre of Polish Mother’s Health, Łódź, Poland

**Keywords:** Phenylketonuria, Adolescents and young adults, Sexual health, Reproductive health, Chronic disease management, Rare diseases

## Abstract

**Background:**

Phenylketonuria (PKU) is a rare inherited metabolic disorder requiring lifelong treatment. While early diagnosis and dietary management have substantially improved neurocognitive outcomes, less is known about how PKU affects sexual life, intimate relationships, and reproductive decision-making, particularly during the transition to adulthood.

**Methods:**

This cross-sectional study included 152 adolescents and young adults with PKU and 183 age-matched healthy controls. A questionnaire-based approach was used to assess sociodemographic characteristics, sexual behavior, and reproductive attitudes. Additional analyses within the PKU group examined sex- and age-related differences and factors associated with awareness of maternal PKU syndrome.

**Results:**

Individuals with PKU differed from controls primarily in psychosocial and reproductive domains rather than in sexual behavior itself. Participants with PKU were more likely to live with parents, less frequently sexually active, and less likely to have children, particularly men. Among sexually active individuals, no significant differences were observed between groups in age at sexual initiation, number of sexual partners, or condom use. Within the PKU cohort, women were more likely to be sexually active, to live independently, to have children, and to report that PKU influenced decisions related to sexual initiation and parenthood. Awareness of maternal PKU syndrome was uneven and strongly associated with female sex, older age, higher education, sexual activity, and parenthood, indicating substantial knowledge gaps among younger individuals and men.

**Conclusions:**

PKU appears to be associated with delayed psychosocial transitions and reduced parenthood rather than altered sexual behavior per se. Marked sex differences in reproductive attitudes and disease-related knowledge highlight an unequal distribution of reproductive responsibility. These findings underscore the need to integrate structured sexual and reproductive health education into lifelong care for individuals with PKU, particularly during adolescence and early adulthood.

## Background

Phenylketonuria (PKU) is an autosomal recessive inborn error of metabolism caused by deficiency of phenylalanine hydroxylase, leading to chronic hyperphenylalaninemia and potential neurotoxicity if untreated [1, 2]. Elevated phenylalanine interferes with cerebral transport of large neutral amino acids and impairs neurotransmitter synthesis, resulting in severe intellectual disability in untreated individuals. The implementation of newborn screening programs has fundamentally changed the natural history of PKU, enabling early diagnosis and prompt initiation of dietary treatment [1]. In Poland, newborn screening for PKU was introduced in 1965, initially covering central regions of the country, and expanded to achieve nationwide coverage by the mid-1980s. PKU is currently included in the national newborn screening programme alongside more than 30 other inherited metabolic disorders, using tandem mass spectrometry (MS/MS). Patients are followed at nine specialised metabolic centres across the country. Dietary treatment based on phenylalanine restriction and phenylalanine-free amino acid supplements is reimbursed through the public healthcare system; however, low-protein specialty foods are not reimbursed. Sapropterin is registered in Poland and available for eligible patients, with reimbursement restricted to specific genotypes [3, 4].

In countries with established newborn screening, early and continuous dietary management effectively prevents severe neurological impairment and allows most individuals with PKU to achieve normal cognitive development. As a result, an increasing number of patients now reach adolescence and adulthood without intellectual disability and participate fully in education, employment, and social life [2, 5, 6]. Consequently, contemporary PKU care has shifted from a primary focus on survival and cognitive outcomes toward long-term health-related quality of life and psychosocial functioning [7–10].

Despite early diagnosis and substantial advances in treatment options, phenylketonuria remains a lifelong condition requiring sustained management, including dietary restriction, regular monitoring of blood phenylalanine levels, and ongoing engagement with specialized healthcare services. Although pharmacological therapies are increasingly available and used in clinical practice, the low-phenylalanine diet remains the most widely applied and foundational component of PKU management worldwide. Adherence to dietary recommendations remains highly variable across the life course and is influenced by multiple factors beyond metabolic control alone. Several studies have demonstrated that difficulties with dietary compliance may emerge already in childhood and intensify during adolescence, coinciding with increasing autonomy and treatment fatigue [3, 11–13]. Importantly, acceptance of the disease and understanding of dietary requirements play a central role in long-term adherence. A multi-centre study by Witalis et al. [14] showed that both patients with PKU and their parents differ substantially in their level of disease acceptance, with acceptance decreasing with age and increasing independence. In a subsequent study, Witalis et al. [15] demonstrated considerable gaps in patients’ and caregivers’ knowledge of dietary principles, as well as variable compliance with dietary recommendations, even among individuals diagnosed and treated from early childhood. These findings indicate that adherence difficulties are not confined to adulthood but may reflect long-standing patterns established earlier in life.

Sexual maturation and reproductive health represent particularly important domains in the transition from pediatric to adult care. While pubertal development in PKU is generally within the normal range, adolescence is also a period associated with increased risk of deteriorating metabolic control [16, 17]. From a clinical perspective, these issues are of particular importance in women with PKU, for whom unplanned pregnancy and inadequate metabolic control carry a substantial risk of maternal phenylketonuria syndrome (MPKU) [18–20]. MPKU is associated with severe teratogenic effects, including congenital heart defects, microcephaly, intellectual disability, and intrauterine growth restriction [21]. Effective contraception, pregnancy planning, and disease-related knowledge are therefore essential components of lifelong PKU care. However, responsibility for reproductive risk prevention may be unevenly distributed between sexes and across age groups, potentially leaving younger individuals and men with insufficient awareness of reproductive consequences. Beyond the well-established teratogenic risks associated with maternal PKU, psychosocial factors may also influence sexual and relational development in both sexes. Subtle deficits in social cognition, including difficulties in interpreting non-verbal cues and complex interpersonal signals, have been documented in early-treated individuals with PKU and may affect confidence and reciprocity in intimate relationships regardless of sex [19]. Furthermore, treatment-related self-consciousness, dietary disclosure anxiety, and prolonged dependence on parental support have been reported in both male and female patients and may contribute to delayed entry into romantic relationships independently of sexual maturation [22, 23]. However, it remains unclear to what extent these well-established clinical risks translate into differences in real-life sexual behaviors, partnership patterns, reproductive outcomes, and disease-related knowledge among individuals with PKU. Despite the complex interplay of biological, psychological, and social factors associated with PKU, empirical data on sexual behaviors, partnership patterns, reproductive outcomes, and disease-related knowledge remain limited. Existing research has predominantly focused on metabolic control and cognitive outcomes, while sexual and reproductive health—and particularly behaviors relevant to the prevention of maternal PKU—have received comparatively little systematic attention. Moreover, sex- and age-specific differences and comparisons with the general population are rarely explored.

Therefore, the aim of this study was to assess differences in sexual behaviors between individuals with PKU and a healthy population, as well as to identify sex-specific differences in these behaviors among patients with PKU, with particular consideration of behaviors relevant to the awareness of maternal phenylketonuria.

## Methods

The study included patients diagnosed with PKU aged 16–35 years recruited from all PKU treatment centers in Poland, as well as a control group of healthy individuals within a comparable age range. All included patients were diagnosed through the national newborn screening programme and initiated dietary treatment in early infancy. Genotype data were not collected in order to preserve participant anonymity, which was a prerequisite of the study’s ethical approval. In the Polish PKU population, the majority of patients are known to present with classical PKU [24]. The control group was recruited during visits to primary care physicians by members of the research team, with the aim of matching the PKU cohort by sex and age. Eligibility criteria for controls included age 16–35 years, absence of any diagnosed metabolic disorder, and no first- or second-degree family relationship with PKU patients. A diagnostic survey method was applied using a questionnaire-based approach. The research instrument consisted of an original questionnaire developed by the Polish Society for Phenylketonuria. The tool incorporated selected questions from the standardized Youth Risk Behavior Survey (YRBS) for adolescents and older youth (2019 National Youth Risk Behavior Survey; 2019 State and Local Standard High School YRBS) [25].

The study was approved by the Bioethics Committee of the Medical University of Białystok (UMB R-I- 002/497/2019). In accordance with the Declaration of Helsinki, all participants provided written informed consent prior to completing the questionnaire [26].

Statistical analyses were performed using JASP (version 0.18.3). Descriptive statistics were used to summarize the sociodemographic, sexual, and reproductive characteristics of the study population. Continuous variables are presented as means with standard deviations (SD), while categorical variables are expressed as numbers and percentages. Comparisons between patients with PKU and the control group were conducted using the χ² (chi-square) test for categorical variables and Fisher’s exact test, as appropriate. When applicable, analyses were additionally stratified by sex and age subgroups. Age was recorded using predefined categories (16–18, 19–25, and ≥ 26 years), reflecting developmentally distinct phases: mid-to-late adolescence, early adulthood, and established adulthood, consistent with established frameworks for the classification of developmental stages in young people [27, 28]. Only questionnaires completed in full were included in the statistical analysis; surveys with unanswered items were excluded.

Sex-specific analyses within the PKU group were performed to assess differences between female and male patients in sociodemographic characteristics, sexual activity, reproductive outcomes, and perceptions of disease impact. Associations between knowledge of MPKU and selected demographic, educational, and clinical factors were also evaluated using χ² tests. All statistical tests were two-tailed, and a p value < 0.05 was considered statistically significant.

## Results

The study included 335 participants, comprising 152 patients with PKU and 183 individuals in the control group (see Table 1). The overall sample was predominantly female (54.6%), and no significant differences were observed between groups with respect to sex distribution, age categories, occupational status, educational level, or place of residence.

Significant between-group differences were identified in family and sexual life characteristics. Patients with PKU were more likely to live with parents or a single parent than controls (66.4% vs. 50.8%, *p* = 0.002) and were less likely to live with a spouse or partner. A markedly higher proportion of PKU patients reported never having engaged in sexual intercourse compared with controls (37.9% vs. 19.1%, *p* < 0.001), while controls more frequently reported sexual initiation at or before 19 years of age.

PKU patients were also more likely to report not having a current life partner (32.9% vs. 14.2%). Among those in relationships, meeting a partner through friends or at school/work was most common in both groups, although meeting partners in nightclubs was less frequent among PKU patients. Parenthood was less prevalent among PKU patients than among controls (21.1% vs. 35.5%, *p* = 0.004), whereas planned age for having children and frequency of social meetings outside school or work did not differ significantly between groups.

**Table 1:** Sociodemographic and familial characteristics of patients with PKU and controls

Characteristics		All participants	Control group	PKU patients	*p* value
Number, *n*		**335**	**183**	**152**	
**Gender**,** years**,** n (%)**					
	Female	183 (54.6)	93 (50.8)	90 (59.2)	ns
	Male	152 (45.4)	90 (49.2)	62 (40.8)	
**Age groups**,** years**,** n (%)**					
	16–18	86 (25.7)	42 (22.9)	44 (29.0)	ns
	19–25	93 (27.8)	51 (27.9)	42 (27.6)	
	≥ 26	156 (46.5)	90 (49.2)	66 (43.4)	
**Occupational status**,** n (%)**					
	Student	143 (42.7)	78 (42.6)	65 (42.8)	ns
	Employed	153 (45.7)	85 (46.5)	68 (44.7)	
	Unemployed	39 (11.6)	20 (10.9)	19 (12.5)	
**Educational level**,** n (%)**					
	Higher education	89 (26.6)	55 (30.0)	34 (22.4)	ns
	Secondary education	138 (41.2)	77 (42.1)	61 (40.1)	
	Primary education	108 (32.2)	51 (27.9)	57 (37.5)	
**Place of residence**,** n (%)**					
	Rural	125 (37.3)	65 (35.5)	60 (39.5)	ns
	Town with 10,000 to 100,000 inhabitants)	113 (33.7)	57 (30.2)	56 (36.8)	
	City with > 100, 000 inhabitants)	97 (29.0)	61 (33.3)	36 (23.7)	
**Living arrangement**,** n (%)**					
	Living with parents/ one parent	191 (57.0)	90 (50.8)	101 (66.4)	0.002
	Living with spouse or partner	144 (43.0)	93 (49.2)	51 (33.6)	
**Age at first sexual intercourse**,** lifetime**,** n (%)**					
	Never	93 (27.8)	35 (19.1)	58 (37.9)	< 0.001
	≤ 19	144 (43.0)	92 (50.3)	52 (34.0)	
	≥ 20	98 (29.2)	56 (30.6)	42 (28.1)	
**Place of meeting current life partner**,** n (%)**					
	No partner	76 (22.7)	26 (14.2)	50 (32.9)	< 0.001
	School/workplace	63 (18.8)	43 (23.5)	20 (13.2)	
	Through friends or neighbors	97 (29.0)	53 (29.0)	44 (28.9)	
	Online	34 (10.1)	17 (9.3)	17 (11.2)	
	Nightclub	25 (7.5)	20 (10.9)	5 (3.3)	
	Other circumstances	40 (11.9)	24 (13.1)	16 (10.5)	
**Parenthood status**,** n (%)**					
	Yes	97 (29.0)	65 (35.5)	32 (21.1)	0.004
	No	238 (71.0)	118 (64.5)	120 (78.9)	
**Planned age for having a child**,** n (%)**					
	No plans	68 (20.3)	44 (24.1)	24 (15.8)	ns
	Before 25 years	70 (20.9)	31 (16.9)	39 (25.7)	
	25–30 years	137 (40.9)	76 (41.5)	61 (40.1)	
	≥ 30 years	60 (17.9)	32 (17.5)	28 (18.4)	
**Meetings with peers outside school/work**,** n (%)**					
	Daily	55 (16.4)	25 (13.7)	30 (19.7)	ns
	1–3 times per week	199 (59.4)	106 (57.9)	93 (61.2)	
	Onse a month or less	81 (24.2)	52 (28.4)	29 (19.1)	

ns – not significant

Table 2 compares sexually active individuals with PKU and those in the control group. Among all participants, 242 individuals reported being sexually active, including 148 controls and 94 patients with PKU. In the overall study population, females (183/335) and males (152/335) were similarly represented, with no significant differences between groups. However, among sexually active participants, a significantly lower number of sexually active males was observed in the PKU group compared with controls (31 vs. 83; *p* < 0.001), while the number of sexually active females was comparable between groups. The age distribution of sexually active participants did not differ significantly between PKU patients and controls. Most participants were aged ≥ 26 years, followed by those aged 19–25 years, with the smallest proportion in the 16–18-year age group. The age at first sexual intercourse was similar in both groups, with the majority reporting initiation before the age of 19 years. The threshold of 19 years was selected to reflect the transition from adolescence to adulthood, consistent with the age categories used elsewhere in the study.

No significant differences were observed between PKU patients and controls with respect to the number of lifetime sexual partners. In both groups, having one sexual partner was the most frequently reported pattern, followed by four or more partners. It should be noted that the ≥ 4 partners category encompasses a wider numerical range than the single-value categories of 2 or 3 partners, which may account for its relatively higher frequency. Condom use was high and comparable across groups, with more than 80% of sexually active participants reporting condom use. Among sexually active women, nearly half reported having a gynecological consultation prior to first sexual intercourse. The proportion of women reporting such a consultation did not differ significantly between patients with PKU and controls.

**Table 2:** Comparison of sexually active individuals with PKU and controls

Characteristics		All Participants	Control Group	PKU patients	*p* value
Number, *n*		242	148	94	
**Gender**, ***n*** **(%)**					
	All Female	183	93	90	ns
	**Sexually active female**	128	65	63	
	All Male	152	90	62	< 0.001
	**Sexually active male**	114	83	31	
**Sexually active participants**					
		**All Participants**	**Control Group**	**PKU patients**	
**Age groups**,** years**,** n (%)**					
	16–18	25 (10.3)	20 (13.5)	5 (5.3)	ns
	19–25	68 (28.1)	39 (26.4)	29 (30.9)	
	≥ 26	149 (61.6)	89 (60.1)	60 (63.8)	
**Age at first sexual intercourse**,** lifetime**,** n (%)**					
	< 19	144 (59.5)	92 (62.2)	52 (55.3)	ns
	≥ 20	98 (40.5)	56 (37.8)	42 (44.7)	
**Number of sexual partners**,** n (%)**					
	1	95 (39.3)	54 (36.5)	41 (43.6)	ns
	2	49 (20.2)	32 (21.6)	17 (18.1)	
	3	41 (16.9)	26 (17.6)	15 (16.0)	
	≥ 4	57 (23.6)	36 (24.3)	21 (22.3)	
**Condom use**,** n (%)**					
	Yes	200 (82.6)	122 (82.4)	78 (83.0)	ns
	No	42 (17.4)	26 (17.6)	16 (17.0)	
**Women (** ***n*** ** = 128)**					
**Gynecological consultation prior to first sexual intercourse**, **n (%)**					
	Yes	61 (47.7)	27 (41.5)	34 (54.0)	ns
	No	67 (52.3)	38 (58.5)	29 (46.0)	

ns – not significant

Table 3 presents a comparison of sociodemographic, reproductive, and sexual characteristics between female and male patients with PKU (*n* = 152). The distribution of age groups, occupational status, educational level, and place of residence did not differ significantly between sexes. Male patients were significantly more likely to live with parents or a single parent compared with female patients (*p* = 0.008), while a significantly higher proportion of women reported having children compared with men (*p* = 0.002). Regarding sexual history, a greater proportion of male patients reported never having had sexual intercourse compared with females, whereas females more frequently reported sexual initiation before the age of 19 years. The distribution of planned age for having a child did not differ significantly between women and men.

**Table 3:** Sociodemographic, reproductive, and sexual characteristics of female and male patients with PKU

Characteristics		PKU patients, *n* = 152		*p* value
		Female	Male	
Number, n		90	62	
**Age Subgroup**,** years**				
	16–18	24 (26.4)	20 (32.2)	ns
	19–25	21 (23.1)	21 (33.9)	
	≥ 26	45 (50.5)	21 (33.9)	
**Profession**,** n (%)**				
	Student	34 (37.3)	31 (50.0)	ns
	Employed	42 (46.2)	27 (43.5)	
	Unemployed	14 (16.5)	4 (6.5)	
**Educational level**,** n (%)**				
	Higher education	24 (26.4)	10 (16.1)	ns
	Secondary education	37 (41.7)	24 (38.7)	
	Primary education	29 (31.9)	28 (45.2)	
**Place of residence**,** n (%)**				
	Rural	38 (42.9)	22 (35.5)	ns
	Urban (city with 10,000 to 100,000 inhabitants)	35 (38.4)	21 (33.9)	
	Urban (city with > 100, 000 inhabitants)	17 (18.7)	19 (30.6)	
**Living situation**,** n (%)**				
	Living with parents/ one parent	52 (58.2)	49 (79.0)	0.008
	Living with spouse or partner	38 (41.8)	13 (21.0)	
**Do you have children?**,** n (%)**				
	Yes	26 (28.9)	5 (8.1)	0.002
	No	64 (71.1)	57 (91.9)	
**Planned age for having a child**,** n (%)**				
	No plans	14 (15.6)	10 (16.1)	ns
	Before 25 years	27 (30.0)	12 (19.4)	
	25–30 years	34 (37.7)	27 (43.5)	
	≥ 30 years	15 (16.7)	13 (21.0)	
**Age at first sexual intercourse**,** lifetime**,** n (%)**				
	Never	27 (30.0)	31 (50.0)	0.044
	≤ 19	35 (38.9)	17 (27.4)	
	≥ 20	28 (31.1)	14 (22.6)	

ns – not significant

Responses to sexual and reproductive life–related questions among patients with PKU are summarized by sex and age subgroup (see Table 4). Women more often than men reported that the disease influenced decisions related to initiating sexual intercourse (44.4% vs. 19.4%, *p* = 0.002) and having children (77.8% vs. 32.3%, *p* < 0.001). No significant sex-related differences were observed for perceived effects of the disease on sexual behavior overall or opinions on informing a partner before sexual intercourse.

Age-related differences were observed in partner awareness and disclosure. Participants aged ≥ 26 years more frequently reported that their partner was aware of their diagnosis and more often agreed that a partner should be informed before initiating sexual intercourse, compared with younger age groups (*p* < 0.001 and *p* = 0.002, respectively). Perceived effects of PKU on sexual behavior, initiation of sexual intercourse, and reproductive decision-making did not differ significantly across age subgroups.

**Table 4:** Sexual and reproductive life–related questions according to sex and age subgroups among patients with PKU

PKU-related questions		Gender		*p*	Age subgroups, years			*p* value
		Female	Male		16–18	19–25	≥ 26	
Number, n (%)		90	62		44	42	66	
**Do you think that your disease affects your sexual behavior?**								
	Yes	26 (29.0)	8 (12.9)	ns	5 (11.4)	14 (33.3)	15 (22.7)	ns
	No	59 (65.5)	50 (80.6)		34 (77.2)	26 (61.9)	49 (74.3)	
	I don’t know	5 (5.5)	4 (6.5)		5 (11.4)	2 (4.8)	2 (3.0)	
**Do you think that your disease affects the decision to initiate sexual intercourse?**								
	Yes	40 (44.4)	12 (19.4)	0.002	15 (34.1)	13 (31.0)	24 (36.4)	ns
	No	50 (55.6)	50 (80.6)		29 (65.9)	29 (69.0)	42 (63.6)	
**Does your partner know that you have PKU?**								
	Yes	62 (68.9)	40 (64.5)	ns	19 (43.2)	33 (78.6)	50 (75.8)	< 0.001
	No	28 (31.1)	22 (35.5)		25 (56.8)	9 (21.4)	16 (24.2)	
**Should a partner be informed about your disease before initiating sexual intercourse?**								
	Yes	65 (72.2)	36 (58.1)	ns	20 (45.4)	31 (73.8)	50 (75.8)	0.002
	No	25 (27.8)	26 (41.9)		24 (54.6)	11 (26.2)	16 (24.2)	
**Do you think that your disease affects the decision to have a child?**								
	Yes	70 (77.8)	20 (32.3)	< 0.001	21 (47.7)	27 (64.3)	43 (65.2)	ns
	No	20 (22.2)	42 (67.7)		23 (52.3)	15 (35.7)	23 (34.8)	

ns – not significant

Awareness of MPKU differed significantly across several sociodemographic and clinical characteristics (see Table 5). Women were more likely than men to report knowledge of the syndrome (70.3% vs. 29.7%, *p* < 0.001). Higher awareness was also observed among older participants, particularly those aged ≥ 26 years, compared with younger age subgroups (*p* = 0.009).

Participants with higher educational attainment more frequently reported awareness of MPKU than those with secondary or primary education (*p* = 0.044). Awareness was also significantly more common among individuals who had children (27.0% vs. 4.9%, *p* = 0.001) and among those who believed that their disease influenced decisions about having children (66.7% vs. 41.5%, *p* = 0.003). In addition, sexually active participants were more likely to be aware of the syndrome compared with those who were not sexually active (72.1% vs. 34.1%, *p* < 0.001).

**Table 5:** Factors associated with awareness of MPKU among patients with PKU

Parameters		Awareness of MPKU		*p* value
		Yes	No	
Number, n		**111**	**41**	
**Gender**				
	Female	78 (70.3)	12 (29.3)	< 0.001
	Male	33 (29.7)	29 (70.7)	
**Age subgroup**,** years**,** n (%)**				
	16–18	25 (22.5)	19 (46.4)	0.009
	19–25	31 (27.9)	11 (26.8)	
	=>26	55 (49.6)	11 (26.8)	
**Educational level**,** n (%)**				
	Higher education	27 (24.3)	7 (17.1)	0.044
	Secondary education	49 (44.2)	12 (29.3)	
	Primary education	35 (31.5)	22 (53.6)	
**Parenthood status**,** n (%)**				
	Yes	30 (27.0)	2 (4.9)	0.001
	No	81 (73.0)	39 (95.1)	
**Perceived impact of the disease on the decision to have a child**,** n (%)**				
	Yes	74 (66.7)	17 (41.5)	0.003
	No	37 (33.3)	24 (58.5)	
**Sexually active**,** n (%)**				
	Yes	80 (72.1)	14 (34.1)	< 0.001
	No	31 (27.9)	27 (65.9)	

ns – not significant

## Discussion

Our study provides a comprehensive analysis of sexual life, partnership relationships, and reproductive issues among adolescents and young adults with phenylketonuria (PKU). The findings indicate that sexual behavior itself, once sexual activity has been initiated, does not differ significantly from that observed in healthy peers. The observed differences primarily concern relational contexts as well as reproductive attitudes, with particularly pronounced sex differences in reproductive decision-making and awareness of maternal phenylketonuria (MPKU) syndrome.

Available literature data indicate that pubertal development in early-treated phenylketonuria generally follows an age-appropriate trajectory; our findings in the present cohort are in line with these observations [16, 18, 29–31]. Earlier studies have also demonstrated no statistically significant differences between individuals with early-treated PKU and healthy controls in achieving core psychosexual developmental milestones. Moreover, earlier work using the Illness Intrusiveness Ratings Scale [18] has shown that sexual life is often perceived as one of the least affected domains of functioning, especially when compared with dietary burden or financial constraints [11, 32–34]. The present findings align with this literature, as sexually active participants reported age at sexual initiation, number of sexual partners, and contraceptive practices comparable to those observed in the general population.

Despite broadly normative sexual behavior, individuals with PKU face distinct psychosocial challenges that may influence intimacy and relationship formation. Adolescence appears to represent a particularly vulnerable period, during which increasing treatment autonomy, fluctuating dietary adherence, and heightened sensitivity to peer norms intersect. Previous qualitative and survey-based studies have highlighted self-consciousness related to dietary management, including concerns about the taste or odor of amino acid supplements, as well as anxiety surrounding disclosure of dietary restrictions in social situations [8, 16, 17, 23, 35–37]. The present findings support this contextual interpretation, suggesting that social experiences related to PKU may contribute to delayed initiation of romantic relationships even in the absence of delayed sexual maturation [18, 38, 39].

The social centrality of food represents a recurring challenge across developmental stages. Earlier studies have noted that dating and social bonding frequently revolve around shared meals, creating situations in which dietary disclosure becomes unavoidable [40, 41]. Women in particular have reported stress related to explaining dietary needs or declining invitations involving food. Although the present study did not explore dating experiences in depth, participants’ responses regarding disclosure and disease awareness are consistent with these previously described barriers, suggesting that PKU-related dietary management continues to shape relational experiences into adulthood.

Importantly, relationship quality was not directly assessed in this study. Nevertheless, partner awareness and disclosure emerged as salient relational dimensions. Most sexually active participants reported that their partner was aware of their diagnosis, and the majority endorsed the view that a partner should be informed about the disease prior to initiating sexual intercourse. These patterns suggest that disclosure and partner awareness are perceived as integral elements of intimate relationships in PKU and may influence how individuals experience and manage the condition. This interpretation is consistent with prior studies highlighting the importance of partner involvement in PKU management [8]. Previous work has demonstrated that shared responsibility for treatment-related tasks, such as dietary planning and meal preparation, is associated with greater relationship satisfaction and emotional stability. Conversely, perceiving PKU as an individual rather than shared responsibility has been linked to increased relational strain [8, 42]. Together, these findings provide a plausible interpretative framework for the present observations and support a relational model of PKU care that extends beyond individual metabolic control.

Sex-specific reproductive concerns emerged as a central theme, particularly among women of reproductive age [18, 30]. The teratogenic risk associated with elevated maternal phenylalanine levels and the resulting MPKU syndrome represents a uniquely burdensome aspect of the disease, directly linking metabolic control with reproductive outcomes. Awareness of MPKU syndrome was uneven and patterned by sex, age, sexual activity, and parenthood status. Women, older participants, and those who were sexually active or had children were more likely to report knowledge of this risk, whereas younger individuals and men demonstrated notable gaps in awareness.

Sex differences were also evident in patterns of sexual activity. Men with PKU were less likely to report being sexually active than women. Importantly, this difference was not accompanied by disparities in age at sexual initiation among those who were sexually active, suggesting that the observed gap reflects delayed or reduced entry into sexual relationships rather than altered sexual development itself. This pattern is consistent with earlier reports indicating that individuals with PKU generally achieve normal psychosexual maturation but may experience difficulties related to self-confidence, social participation, and relationship formation. It should also be noted that the sex distribution across age subgroups within the PKU cohort was unequal, with a higher proportion of women in the ≥ 26 years group and a greater representation of women with higher education. These differences may have partially contributed to the observed disparities in sexual activity between male and female patients and should be considered when interpreting the findings. In men, lower rates of sexual activity may reflect prolonged residence in parental households, fewer partnership opportunities, and lower perceived relevance of reproductive issues rather than intrinsic sexual dysfunction [30, 43]. Together with lower awareness of MPKU syndrome, these findings suggest that men with PKU may enter intimate relationships later and with less perceived disease-related responsibility, reinforcing sex-specific trajectories in sexual and reproductive life [23]. Notably, this pattern represents a reversal of the trend observed in the Polish general population, where men typically initiate sexual activity slightly earlier than women, with median ages of approximately 18.5 and 19 years, respectively [44]. In contrast, within the PKU cohort, women more frequently reported sexual initiation before the age of 19 years (38.9% vs. 27.4% in men), while men were significantly more likely to report never having had sexual intercourse (50.0% vs. 30.0%, *p* = 0.044). This reversal suggests that the observed sex difference may not simply reflect general population trends, but rather a disease-specific pattern in which men with PKU are disproportionately affected in terms of delayed or absent entry into sexual relationships.

Disparities in reproductive knowledge provide important context for the pronounced sex differences observed in reproductive decision-making. Women with PKU were more likely than men to perceive the disease as influencing their decision to have a child, underscoring the disproportionate cognitive and emotional burden borne by female patients in relation to parenthood. This finding aligns with previous studies describing heightened reproductive anxiety among women with PKU, including fear of unplanned pregnancy, strict contraceptive practices, and, in some cases, avoidance of sexual intimacy. While pregnancy is typically associated with positive expectations, for women with PKU it may instead represent a source of anxiety and perceived risk, with potential consequences for relationship formation and emotional well-being. In contrast, men were less likely to perceive PKU as influencing their sexual or reproductive decisions. This pattern may partly reflect lower awareness of reproductive implications rather than absence of concern. Prior studies have documented gaps in knowledge among male patients regarding inheritance patterns and reproductive risk, suggesting that reproductive counseling has historically been more strongly oriented toward women [23]. Although paternal PKU does not confer teratogenic risk when maternal metabolic control is normal, limited understanding of reproductive genetics may nonetheless contribute to asymmetrical perceptions of responsibility within intimate relationships.

Neuropsychiatric factors likely represent an additional layer influencing sexual and relational functioning in PKU. Previous research has demonstrated associations between suboptimal metabolic control and mood disturbances, anxiety, attentional difficulties, and executive dysfunction, even in individuals with preserved global intelligence [16, 17, 22, 33, 37]. Such symptoms are known to affect libido, communication, and emotional regulation and may indirectly shape intimacy and relationship stability [42, 45]. As data on blood phenylalanine concentrations and current treatment adherence were not collected, the relationship between metabolic control and the observed outcomes could not be evaluated directly. Previous studies indicate that suboptimal metabolic control may affect neurocognitive and neuropsychiatric functioning, and could therefore contribute to individual differences in social and reproductive behaviours. Future studies should incorporate longitudinal metabolic data to address this question. The present findings are consistent with the growing recognition that metabolic and cognitive outcomes alone are insufficient to capture the lived experience of adolescents and adults with PKU [18, 23]. In the Polish general population, data from the Health Behaviour in School-Aged Children study indicate a median age at sexual initiation of approximately 18.5 years for boys and 19 years for girls. National survey data reported by Izdebski similarly indicate that the mean age at first intercourse among Polish women has declined to just above 18 years [44, 46]. These figures are broadly consistent with the patterns observed in our control group, suggesting that the control sample reflects general population norms with respect to sexual initiation in Poland. Sexual and reproductive health remains insufficiently addressed in routine metabolic care, particularly during the transition from pediatric to adult services. Many participants appeared to acquire reproductive knowledge reactively rather than proactively, often only after becoming sexually active or considering parenthood. This pattern may leave younger patients insufficiently prepared for informed decision-making and place a disproportionate psychosocial burden on women.

Importantly, beyond mood and executive functioning, prior studies have demonstrated subtle but persistent alterations in social cognition among early-treated individuals with PKU, including difficulties in interpreting non-verbal cues and complex interpersonal signals [19]. Although these deficits are often mild and may not manifest as overt cognitive impairment, they may nonetheless influence confidence, reciprocity, and responsiveness in intimate and sexual relationships. The persistent social–cognitive gap observed between individuals with PKU and unaffected peers has been described as a nuanced burden that may affect competence in social and sexual contexts, even in the absence of measurable intellectual disability [38, 47]. Our findings, showing differences primarily in relational contexts and timing of sexual engagement rather than in sexual behavior itself, are consistent with this framework [20, 21, 30, 31, 48].

In summary, despite largely normative sexual behaviors, adolescents and young adults with phenylketonuria exhibit delayed psychosocial transitions and reduced parenthood, particularly among men. Awareness of MPKU syndrome is uneven and closely linked to sex and life-course stage, leaving younger individuals—especially adolescent girls—at risk of entering intimate relationships without sufficient reproductive knowledge. These findings highlight the need for proactive, age- and sex-specific sexual and reproductive health education integrated into lifelong PKU care, with particular emphasis on early prevention of maternal phenylketonuria.

## Limitations

Several limitations should be considered when interpreting the findings of this study. First, the cross-sectional design precludes causal inference. Observed associations between PKU-related factors, sexual behavior, and reproductive attitudes cannot be interpreted as directional or developmental effects over time. Longitudinal studies would be required to clarify how metabolic control, psychosocial adaptation, and sexual and relational experiences interact across the life course. Second, sexual life and reproductive attitudes were assessed using self-reported measures, which are inherently subject to recall bias and social desirability effects. Given the sensitive nature of sexuality and reproductive decision-making, some participants may have underreported difficulties or tailored responses to perceived social norms, particularly younger respondents.

Third, relationship quality was not directly measured using validated instruments. Interpretations regarding relational functioning are therefore indirect and based on patterns related to disclosure, partner awareness, and perceived disease impact. While these findings are consistent with prior literature emphasizing the role of partner involvement in PKU management, conclusions regarding relationship satisfaction or stability should be regarded as interpretative rather than definitive. Fourth, metabolic control was not assessed longitudinally and could not be linked to specific developmental periods. Although variability in dietary adherence from early childhood onward is well documented in the PKU literature, the present study could not fully capture how fluctuations in metabolic control may have influenced sexual or relational outcomes.

Finally, the study sample may limit generalizability. Participants were recruited from specialized metabolic centers and may represent individuals with relatively higher health literacy and treatment engagement. Individuals disengaged from care, who may experience greater psychosocial burden, could be underrepresented. In addition, the relatively small number of participants with children limited more detailed analyses of parenthood-related outcomes, and male reproductive health was not directly assessed. The living arrangement categories did not include an option for living alone, which may have limited the granularity of data on residential independence. The survey instrument was not subjected to a full psychometric validation process, including cognitive interviewing, concept elicitation, or test-retest reliability assessment. Although the selected YRBS-derived questions address behaviours that remain relevant across the age range studied, the lack of formal validation for use in adult populations represents a methodological limitation. The recruitment strategy for the control group may also have introduced selection bias, as individuals recruited in primary care settings may not be fully representative of the general population. Furthermore, sexual orientation was not assessed, which limits the ability to evaluate its potential contribution to the observed differences between groups. This study was conducted in a single country and the findings may not be generalisable beyond the Polish cultural and healthcare context; multicentre international studies are needed to determine whether the observed patterns persist across different settings. Exploratory stratified analyses of sexual activity among male PKU patients according to educational level and place of residence were conducted but yielded no statistically significant findings; given the limited sample size of this subgroup (*n* = 62 males, of whom only 31 reported being sexually active), these analyses were underpowered and are not reported in detail. Genotype data and newborn screening phenylalanine values were not collected, as combining such information with sociodemographic data would have compromised participant anonymity. The potential influence of disease severity on sexual and reproductive outcomes therefore could not be assessed.

Despite these limitations, this study provides novel insights into sexual life, relational contexts, and reproductive awareness in adolescents and young adults with PKU, highlighting important targets for future research and clinical intervention.

## Conclusions

In conclusion, adolescents and young adults with phenylketonuria exhibit largely normative sexual behaviors once sexual activity is initiated, but experience meaningful differences in the psychosocial and reproductive contexts in which intimacy occurs. Delayed independence, lower rates of partnership formation and parenthood, and substantial variability in reproductive knowledge indicate that the impact of PKU extends beyond metabolic control into key developmental domains associated with the transition to adulthood.

Marked sex differences highlight an unequal distribution of reproductive responsibility, with women more likely to perceive PKU as influencing decisions related to sexual initiation and parenthood, while men demonstrate lower engagement and awareness in these areas. The coexistence of typical sexual development with delayed psychosocial transitions and uneven knowledge of maternal PKU syndrome underscores a critical gap in current models of care.

These findings emphasize the need to integrate structured, age-appropriate, and sex-specific sexual and reproductive health education into routine PKU management, beginning in adolescence and continuing through young adulthood. Addressing these dimensions is essential to support informed decision-making, reduce psychosocial burden, and promote equitable participation in intimate and family life among individuals with PKU. As psychosocial transitions are heavily influenced by regional cultural norms, healthcare systems, and access to treatment, the generalisability of these findings beyond the Polish context should be interpreted with caution. Multicentre international studies are needed to determine whether the observed patterns of relational delay and reproductive knowledge gaps persist across different cultural and healthcare settings.

## Data Availability

No datasets were generated or analysed during the current study.

## Abbreviations

PKU: Phenylketonuria
MPKU: Maternal phenylketonuria
